# Clubfoot treatment with Ponseti method—parental distress during plaster casting

**DOI:** 10.1186/s13018-020-01782-8

**Published:** 2020-07-17

**Authors:** Christian Walter, Saskia Sachsenmaier, Markus Wünschel, Martin Teufel, Marco Götze

**Affiliations:** 1 Orthopedic Department, University Hospital Tübingen, Hoppe Seyler–Str. 3, 72076 Tübingen, Germany; 2 Ortho-Zentrum, Waldstraße 67, 76133 Karlsruhe, Germany; 3 Clinic for Psychosomatic Medicine and Psychotherapy, LVR University Hospital, University Duisburg-Essen, Virchowstraße 174, 45147 Essen, Germany; 4 Clinic for Orthopedics and Trauma Surgery, Heidelberg University Hospital, Schlierbacher Landstraße 200a, 69118 Heidelberg, Germany; 5 Department of Psychosomatic Medicine and Psychoptherapy, University Hospital Tübingen, 72076 Tübingen, Germany

**Keywords:** Parental distress, Club foot, Ponseti method, Pirani score

## Abstract

**Background:**

Clubfoot is one of the most prevalent musculoskeletal congenital defects. Gold standard treatment of idiopathic clubfoot is the conservative Ponseti method, including the reduction of deformity with weekly serial plaster casting and percutaneous Achilles tenotomy. It is well known that parents of children with severe and chronic illnesses are mentally stressed, but in recent studies regarding clubfoot treatment, parents were only asked about their satisfaction with the treatment. Largely unknown is parental distress before and during plaster casting in clubfoot.

Therefore, we want to determinate first, how pronounced the parents’ worries are before treatment and if they decrease during the therapy. Second, we hypothesized that parents faced with an extreme deformity (high Pirani score), reveal more distress, than parents whose children have a less pronounced deformity (low Pirani score). Therefore, we wanted to investigate whether the Pirani score correlates with the parents’ mental resilience in relation to the therapy of the child as a global distress parameter.

**Methods:**

To answer this question, we developed a questionnaire with the following emphases: Physical capacity, mental resilience, motion score, parents score, and child score with point scores 1 (not affected) to 6 (high affected). Subsequently, we interviewed 20 parents whose children were treated with clubfeet and determined the Pirani score of the infants at the beginning (*T*_0_) and at the end (*T*_E_) of the treatment with plaster casting.

**Results:**

High values were obtained in child score (Mean (*M*) = 3.11), motion score (*M* = 2.63), and mental resilience (*M* = 2.25). During treatment, mental resilience improved (*p* = 0.015) significantly. Spearman correlation coefficient between Pirani score (*T*_0_) and mental resilience (*T*_0_) is 0.21, so the initial hypothesis had to be rejected.

**Conclusion:**

The issues of the children are in the focus of parental worries concerning clubfoot treatment, especially the assumed future motion and the assumed ability to play with other children. Particular emphasis should be placed on educating parents about the excellent long-term results in the function of the treated feet especially as this topic shows the greatest parental distress.

## Introduction

Idiopathic clubfoot is one of the most prevalent musculoskeletal congenital defects (1-2 per 1000 live births), which is not self-healing. The deformity will deteriorate until adulthood and cause adverse effects for the patient, if early treatment does not occur [1].

The gold standard for the treatment of idiopathic clubfoot is the conservative Ponseti method [2]. Previous studies had demonstrated better results compared with traditional surgical methods, like the posteromedial release described by Turco [3]. Caused by the Ponseti method, the rate of extensive surgery to treat idiopathic clubfoot decreased substantially [4]. Through this success, the method became accepted all over the world. There is evidence of Ponseti treatment activity in 113 of 193 United Nations [2]. To classify the severity of clubfoot, the Pirani score is one of the most popular classification systems, because it is simple and reliable [5].

The outcome of the children after treatment with the Ponseti method is very well studied, even in long term follow-up. However, the role of the parents in clubfoot deformity has been insufficiently studied. Today, it is well known that an increasingly important source of information is the Internet besides the traditional trust on health care professionals for clubfoot advice [6].

For children with severe and chronic illnesses is confirmed that the parents are mentally stressed [7]. For example, parental distress has been reported in 30–80% of parents with congenital heart disease and 40% report a need for psychosocial care [7].

In terms of clubfoot treatment, parents were only asked about their satisfaction with the treatment [8]. Roye et al. performed a semi-structured qualitative interview asking the parents about, e.g., physical function, pain, and social functioning. Only one question in the questionnaire aims at the mental health of the parents: “What are your feelings about the appearance of your child’s foot?” [9].

Largely unknown are the fears and worries of parents before and during the clubfoot treatment.

The aim of the present study was to determine the state of the mental distress before and after the plaster casting and to work out the main points that will have to be focused on in the advice and support of the parents in the future.

Therefore, we wanted to determinate first, if we could find an improvement in the Pirani score during serial plaster casting in our cohort, according to the recent literature. Second, we developed a clubfoot questionnaire determining parental distress and wanted to investigate the objectivity and reliability. Third, we wanted to investigate, how pronounced the parents’ worries are before treatment, and if they decrease during the therapy. Finally, we hypothesized that parents faced with an extreme deformity (high Pirani score) in their child, reveal more distress, than parents whose children have a less pronounced deformity (low Pirani score). Therefore, we wanted to investigate whether the severity of the child’s deformity (Pirani score) correlates with the parents’ mental resilience in relation to the therapy of the child as a global distress parameter. To answer these questions, we asked 20 parents before and after the plaster casting, whose children were treated with clubfoot in our outpatient clinic, using the questionnaire we developed.

## Patients and methods

### Questionnaire

The questionnaire was developed jointly by all authors. In addition to the treatment and counseling experience of the pediatric orthopedic surgeons, the experience in creating and analyzing questionnaires of the involved psychiatrist was included. In the questionnaire, the practitioners’ known fears of parents from the treatment experience as well as non-articulated, suspected concerns were included.

The following questionnaire structure was decided: First, we asked the parents about their own physical capacity and mental resilience in relation to the therapy of the child in a global statement. The participants were able to give a point value from 1 (not limited) to 6 (highly limited) (see Table 1: statement).

**Table 1:** Clubfoot questionnaire emerged from our orthopedic and psychiatric experience. The questionnaire was translated into the English language for publication

Nr.	Statement	Not limited			Highly limited		
1	Own current physical capacity (in relation to the therapy of the child)	1	2	3	4	5	6
2	Own current mental resilience (in relation to the therapy of the child)	1	2	3	4	5	6
	Parents Score	Not at all			Very strong		
1	My partnership is burdened by the deformity.	1	2	3	4	5	6
2	My spare time activities are limited by the deformity.	1	2	3	4	5	6
3	My finances are limited by the deformity.	1	2	3	4	5	6
4	My professional life is limited by the deformity.	1	2	3	4	5	6
	Motion Score	Not at all			Very strong		
1	The movement of my child will be limited.	1	2	3	4	5	6
2	Playing with other children will be limited.	1	2	3	4	5	6
	Child Score	Not at all			Very strong		
1	My child will not be accepted by others.	1	2	3	4	5	6
2	My child will be restricted in choosing a career.	1	2	3	4	5	6
3	My child will have a hard time at school.	1	2	3	4	5	6
4	My child will be teased.	1	2	3	4	5	6

Second, we were interested in the environment of the parents (parents score). In detail, we asked about limitations in partnership, spare time, finances, and professional life (see Table 1: parents score). In the further questionnaire, the parents were able to tick a point value from 1 (not at all) to 6 (very strong).

Third, we wanted to determine the fears and worries of the parents concerning the future motion of the child (motion score). In detail, we asked about the feared limitation of movement and limitations in playing with other children (see Table 1: motion score).

Finally, we wanted to inquire the parents’ concerns about the child’s global development. This meant in detail the question of the acceptance by other children, the restriction in choosing a career, having a hard time at school, or being teased by other children (see Table 1: child score). At the end, the parents had the possibility to add own concerns in a free text field.

To evaluate the questionnaire, the statements on physical capacity and mental resilience were analyzed individually. Furthermore, in the three scores (parent score, motion score, and child score), the answers of one questionnaire were averaged for further analysis.

The questionnaire was given to the parents in written form and could always be carried out in a quiet corner of the treatment room.

### Study population

**Fig. 1:**
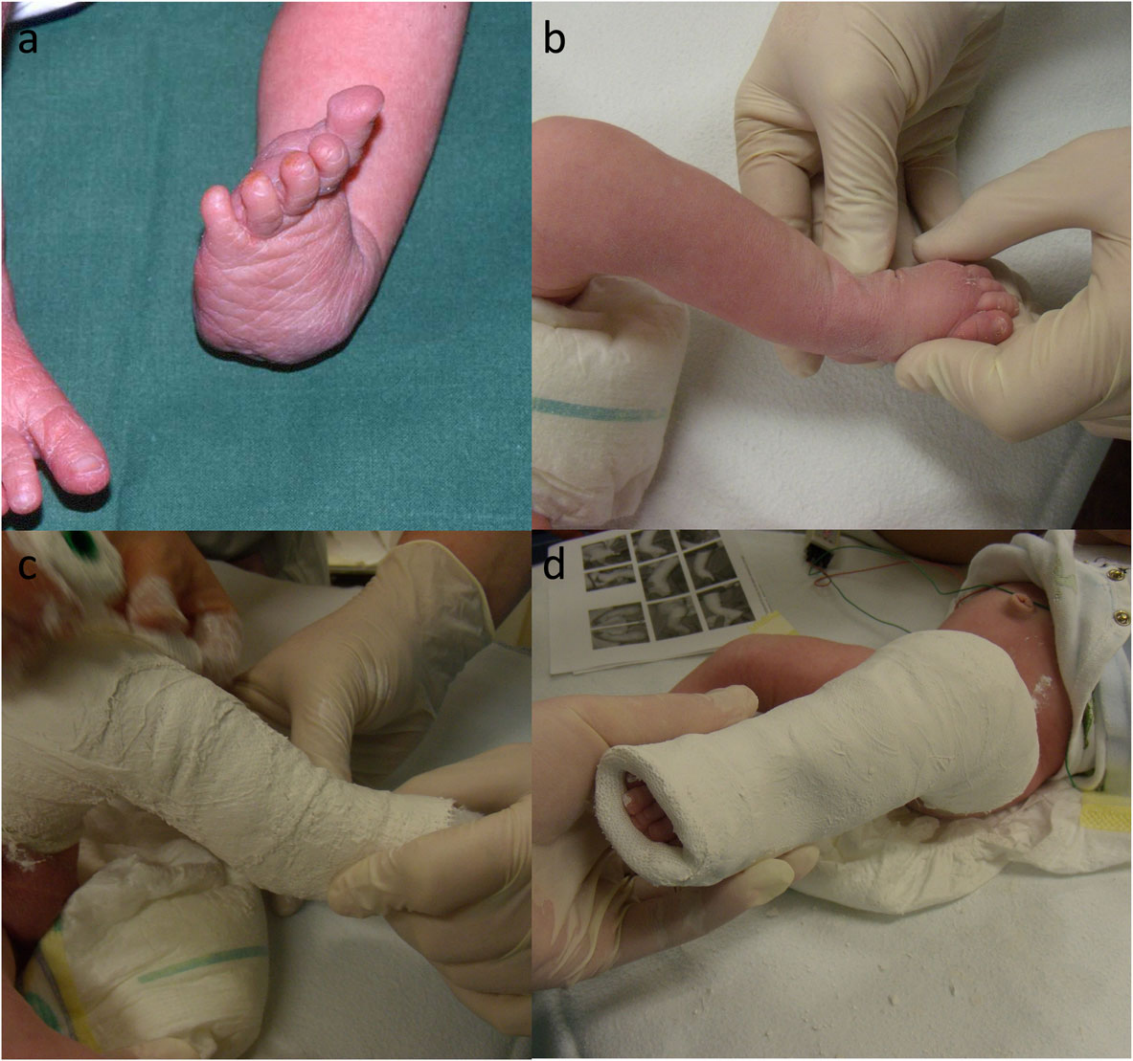
**a** Clubfoot deformity with adduction, equines, and supination. The cavus foot component (pes excavatus) is not visible in this clinical picture. **b** Manipulation position, which is held for approximately 1 min (**c**) applying the plaster (**d**) finished plaster

Twenty study participants whose children/adoptive children were treated for clubfeet (see Fig. 1a) in our outpatient clinic with the Ponseti method were included in the study. Treatment of the child with plaster casting in our outpatient clinic due to irredressible clubfoot and being available for the entire treatment period of the child were inclusion criteria for the parents. Further, we only included parents whose children started treatment at least 10 days after birth. All participants gave written informed consent. Parents with pre-existing mental illness requiring treatment or participating in other clinical trials to test pharmaceuticals, biological substances, or medical devices were excluded from the study as well as parents, whose children had a redressable clubfoot position and did not need plaster casting.

Prior to the study, all parents were thoroughly informed about the expected course of treatment by reducing the deformity weekly with serial plaster casting, the requirement for percutaneous Achilles tenotomy in most cases, and the preservation of the correction with braces. For ethical reasons, parents also had to be informed about the high success rate of the Ponseti method (up to 98%) before filling in the first questionnaire.

After the diagnosis of “clubfoot” in the child, empirical data of the parents (study participants) was requested by the questionnaire. We asked about relationship with the treated child (physical or adopted), specifics during pregnancy and birth, other illnesses of the child, other children with the same illness, pre-treatment information (internet, relatives/friends, books, others), and pre-existing mental illness.

Subsequently, we determined the Pirani score, interviewed the parents using our clubfoot questionnaire (see Table 1) (*T*_0_), and started with the serial plaster casting described by Ponseti.

The treatment started for all study participants within 10 days after birth.

### Plaster casting procedure

The children were calmed as far as possible before manipulation and plastering (e.g., by breastfeeding). All manipulations and plaster castings were carried out by two persons (first and second author). First, the cavus foot was corrected by supination of the forefoot until the plantar surface of the foot shows a normal longitudinal arch. Second, we carried out the manipulation: For this, the talus head is localized and then stabilized with the thumb. Then, we performed the abduction of the foot in supination as far as the child tolerated and held this position for about 1 min (see Fig. 1b).

Finally, we put on the plaster: To do this, we wrapped the foot up to the knee joint with a thin layer of cotton wool. To apply the plaster, the forefoot is held with the index finger and thumb. The plaster must be modeled over the talus head as long as the foot is held in the corrected position (see Fig. 1c). Finally, the plaster is molded, left long on the sole of the foot to support the toes, and shortened dorsally to the metatarsophalangeal joints (see Fig. 1d).

The plaster casting was repeated weekly until the indication for Achilles tenotomy was given. Before applying the final plaster, the clubfoot questionnaire was handed over, and the Pirani score was determined again (*T*_E_).

### Data analysis

The questionnaires were handed over to the study participants in paper form and, after being answered, transferred into Excel. Further analyses were performed using SPSS-Statistics (IBM, Version 25.0.0.1).

For group comparison, we performed the non-parametric Wilcoxon test due to the lack of normal distribution (*p* > 0.05 in the Shapiro-Wilk test) and data outliners, as well as the Bonferroni method for adjusting *p* values in multiple testing (adjusted *p* value (*p*_adj_) = *p* value (*p*) × Number of tests).

For reliability analysis, Cronbach’s alpha [10] was calculated to assess the internal consistency of the five groups (exercise capacity, mental resilience, parents score, motion score, child score). The level of significance was chosen at *p* < 0.05.

## Results

Due to the close bond between the parents and us as the health care professionals during the serial casting, we had no dropouts during the study. All study participants were biological parents and in all children, the diagnosis clubfoot was given immediately after birth; therefore, treatment started on average 3 days after birth. In two cases, there were abnormalities during pregnancy (pelvic vein thrombosis, gestational diabetes). None of the study participants had other children with clubfoot. The previous knowledge of the participants was inconsistent: 37.5% had no prior information, 50% had informed themselves on the Internet, and 12.5% had advance information from friends and acquaintances. An average of 5.9 plaster treatments was required. During the entire study, there were no entries in the free text field described above.

**Fig. 2:**
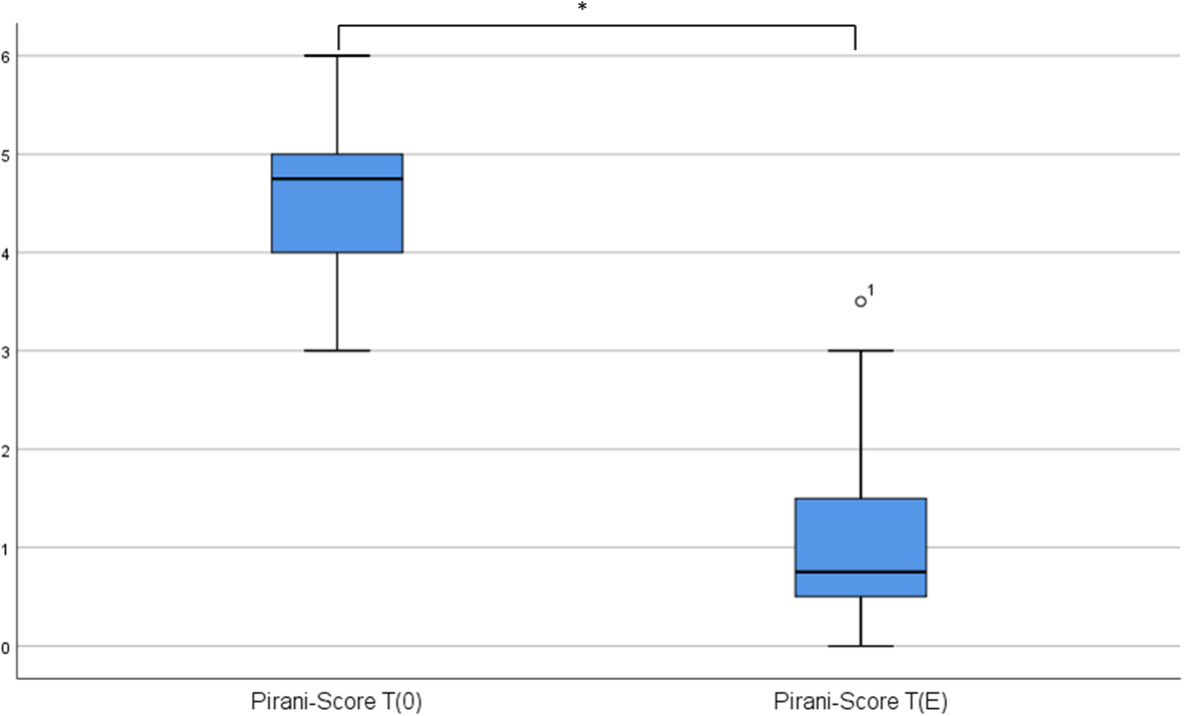
Pirani-Score before (*T*_0_) and after (*T*_E_) treatment with serial plaster casting. Score values strongly decrease during serial plaster casting. Asterisk denotes a significant difference (*p* < 0.05) after Wilcoxon test

First, we asked if there is an improvement of the Pirani score during serial plaster casting. We therefore compared the values of the Pirani score at the beginning (*T*_0_) and at the end (*T*_E_) of the treatment (see Fig. 2). As expected, we found a decrease of 4 points of the Pirani score from *T*_0_ (Median (MED): 4.75 points, interquartile range (IQR): 0.88) to *T*_E_ (MED: 0.75 points, IQR: 0.88) with a significant effect (Wilcoxon test: *z* = −2.98, *p* = .005, *n* = 10). The effect size (*r* = *z*/√*n*) according to Cohen was *r* = .94 and corresponded to a strong effect.

Second, we asked if our clubfoot questionnaire is objective and reliable. The questionnaire is structured in a standardized manner and the instructions for answering were clear. Therefore, the implementation objectivity is secured. The evaluation objectivity was ensured by using closed answers, as well as the interpretation objectivity by specifying exact point scores.

The internal consistency of the questionnaire is high, with Cronbach’s alpha = 0.83. In summary, we were able to demonstrate a high level of objectivity and reliability for our clubfoot questionnaire.

Third, we wanted to investigate, how pronounced the parents’ worries are before treatment. To answer this question, we summed up the point scores marked by the parents for each individual statement and created a ranking. The aim of this ranking was to find out which individual statement achieves particularly high scores and thus high parental distress. The highest total score by far, we found in the statement: “The movement of my child will be limited.” All other statements evoked fewer worries (see Table 2). All in all, there is a clear trend: Statements that affect the concerns of the children were given significantly higher point scores than statements that affect the parental concerns.

**Table 2:** Total score values of our clubfoot questionnaire before plaster casting (*T*_0_) (minimum: 20 points (no stress); maximum: 120 points, (very high stress))

Nr.	Ranking	Total score
1	The movement of my child will be limited.	61
2	Own current psychological resilience (in relation to the therapy of the child)	45
3	Playing with other children will be limited.	45
4	My child will be restricted in choosing a career.	42
5	My child will have a hard time at school.	41
6	My child will be teased.	41
7	My child will not be accepted by others.	40
8	Own current physical capacity (in relation to the therapy of the child)	35
9	My finances are limited by the deformity.	33
10	My spare time activities are limited by the deformity.	32
11	My professional life is limited by the deformity.	31
12	My partnership is burdened by the deformity.	27

**Fig. 3:**
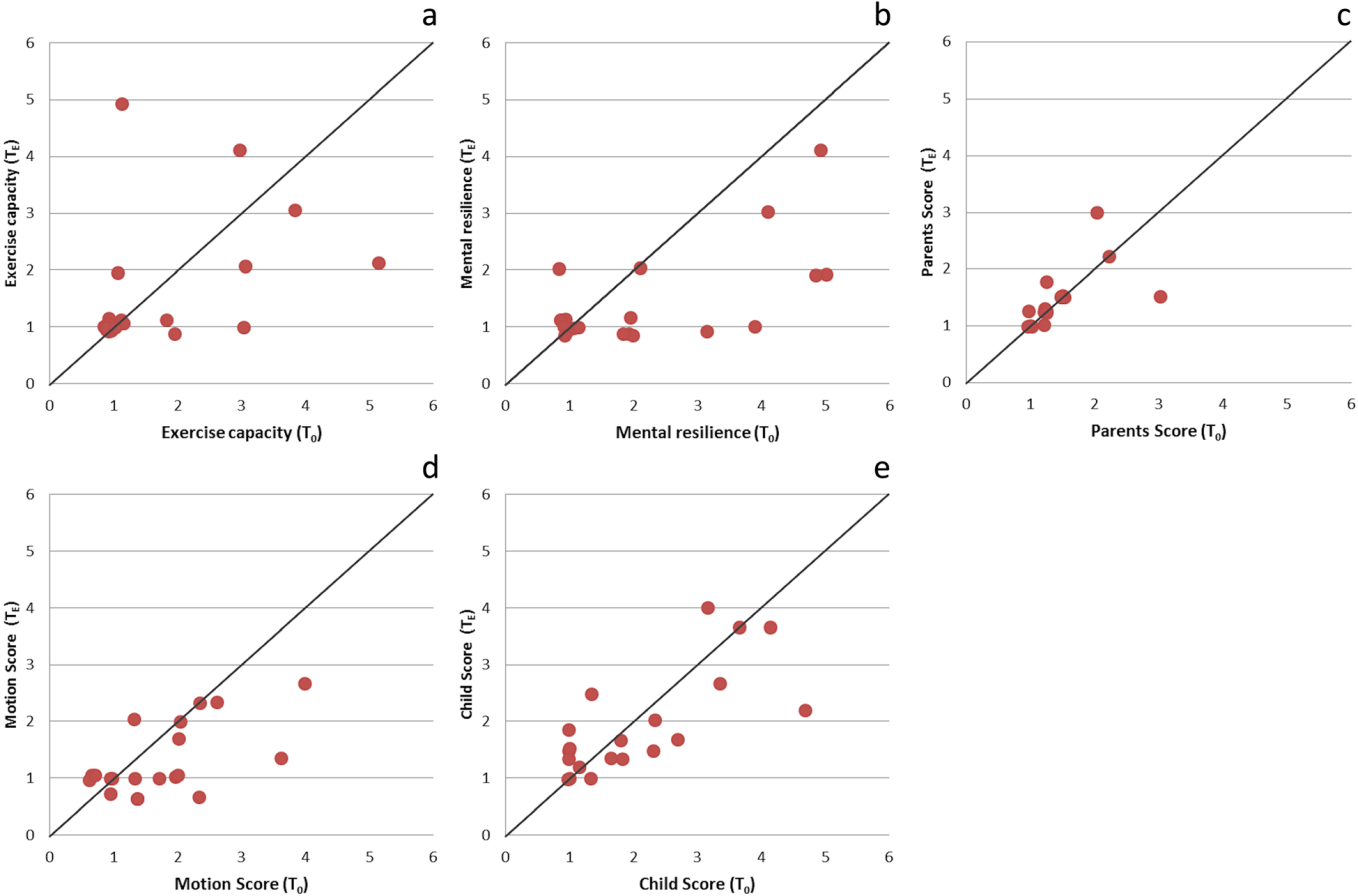
Jitter plots: The *x*-axis shows the scores at the beginning of the treatment (*T*_0_), the *y*-axis at the end (*T*_E_). Data points below the black line show less distress (decrease in the point scores) during treatment, data points above the line more distress (increase of point scores) and data points on the black line show no change. In mental resilience (*p* = 0.04) (**b**) a significant decrease in point score was observed

Subsequently, we want to determine the development of the worries and asked if they decrease during treatment. The background of this question was the assumption that parents’ concerns decrease during treatment when they see the clinical improvement in deformity. We therefore compared the average score values of the five scores (exercise capacity, mental resilience, parents score, motion score, and child score) at the beginning (*T*_0_) and at the end of serial plaster casting (*T*_E_). The level of the point values and their development during treatment is constituted in Fig. 3. Data points at the bottom left of the graph show a low score with little distress; top right a high score with high distress. Data points below the black line show a positive course during treatment (decrease in distress); points above the black line show a negative course (increase in distress). The more distance between line and point the larger is the difference between *T*_0_ and *T*_E_.

In order to analyze whether there is a statistically significant difference between *T*_0_ and *T*_E_, we first checked the normal distribution of the data. The difference of point values (*T*_E_−*T*_0)_ was normally distributed (Shapiro-Wilk test < 0.05), but outliers were found in the data.

As pointed out in Fig. 3b, with the majority of the data points below the black line, we found a significant decrease in mental resilience from *T*_0_ (Mean (*M*) = 2.25, Standard deviation (SD) = 1.51) to *T*_E_ (*M* = 1.45, SD = 0.80), (Wilcoxon test: *z* = −2.658, *p* = .008, *p*_adj_ = .04, *n* = 20). The effect size according to Cohen was *r* = 0.59 and corresponded to a medium effect.

Further, we found a decrease in the analysis of the motion score (see Fig. 3d) from *T*_0_ (*M* = 2.63, Standard deviation (SD) = 1.39) to *T*_E_ (*M* = 1.95, SD = 0.94), but without statistical significance after adjusting the *p* value (Wilcoxon test: *z* = −2.432, *p* = 0.015, *p*_adj_ = 0.075, *n* = 20).

In the exercise capacity (see Fig. 3a) and the child score (see Fig. 3d), we found only a very small decrease of score values (***EC***, *T*_0_*M* = 1.7 to *T*_E_*M* = 1.6; ***CS***, *T*_0_*M* = 3.11 to *T*_E_*M* = 2.89). No changes were found in parents score (*T*_0_ and *T*_E_, *M* = 1.44, see Fig. 3c). In conclusion, we can see clear decreases in mental resilience and motion scores during treatment.

**Fig. 4:**
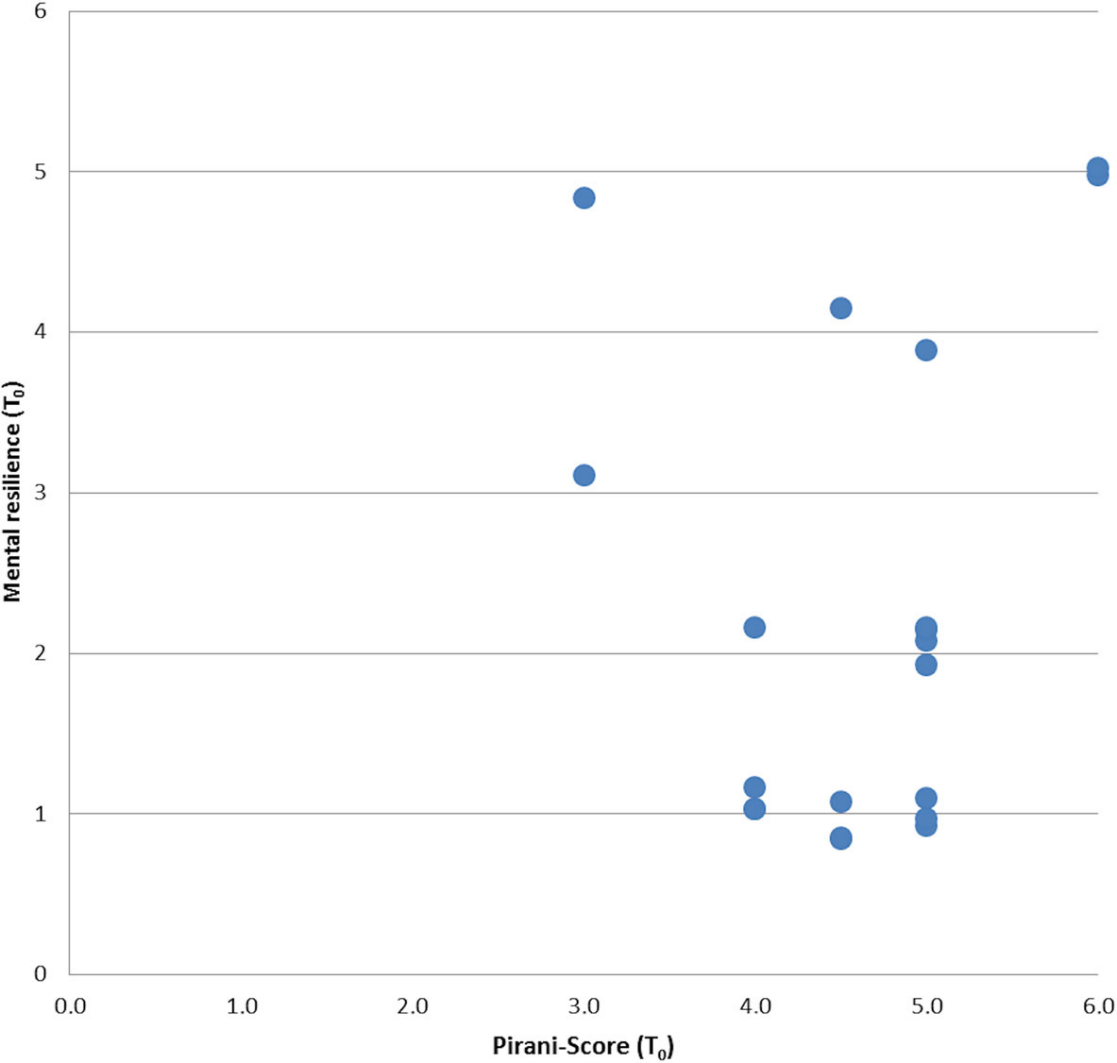
Jitter plot: Comparison between severity of deformity (Pirani score) and level of mental resilience at the beginning of treatment. We found no significant correlation in our cohort (correlation coefficient according to Spearman = 0.21)

Finally, we wanted to investigate whether the parents’ mental resilience correlates with the severity of the child’s deformity (Pirani score). We have therefore calculated the correlation coefficient according to Spearman between mental resilience at time *T*_0_ with the initial Pirani score (*T*_0_) (see Fig. 4) and found no correlation (*r* = 0.21, *p* = 0.365). The initial hypothesis that parents faced with an extreme deformity (high Pirani score) in their child, reveal more distress, than parents whose children have a less pronounced deformity (low Pirani score) had to be rejected.

## Discussion

In this study, we could show as a key result that the issues of the children are in the focus of parental worries concerning clubfoot treatment, especially the assumed future motion and the assumed ability to play with other children. Further, we found a decrease of the worries in the course of treatment in line with the visible improvement in the clinical findings. Finally, we can report that the severity of parents’ concerns is independent of the severity of clubfoot.

In our study, we were able to prove the success of the Ponseti method by the highly significant improvement in the Pirani score, like many authors before us. Due to the overwhelming success, treating clubfoot using the Ponseti method is essential for us. However, from a physiotherapeutic point of view, there are opinions that criticize the immobilization in the plaster. An alternative treatment is the French functional method consisting of daily manipulations of the newborns’ clubfoot and temporary immobilization of the foot with nonelastic adhesive strapping [11]. In experienced hands, this method achieved slightly inferior but comparable results [12]. From a pathoanatomical point of view, we see immobilization as indispensable for remodeling the malformed structures of the tarsal bones [13], so that we only use the Ponseti method in our outpatient clinic.

In our study, we used the Pirani score as a target parameter to describe the severity of deformity, consisting of midfoot and hindfoot contracture part, and can range from 0 to 6 points [14]. However, this classification is not to be viewed without criticism. Other classifications were, e.g., described by Harrold and Walker [15], with a focus on the ability to correct the deformity. The Catterall system [16] is focused on the evolution of deformity, and Diméglio [17] presented the most detailed scoring system with a scale of 0-20. In a comparison of the classifications by Wainwright et al., the Diméglio system showed the highest reliability [18]. However, the authors conclude that current classification systems for the analysis of congenital clubfoot are not entirely satisfactory. In a previous study by Agarwal et al. has examined the influence of the Pirani score on the number of casts and found a positive correlation between the parameters [19]. Dyer et al. describe the Pirani score as reliable and easy to use with a good forecast about the expected treatment [5]. Therefore, we decided to use the Pirani score in our study. Comparing the determined values for the Pirani score at the beginning of treatment, we found similar results (our results: 4.75, Fan et al.: [1] 4.4, Agarwal et al.: [19] 5.0).

The central topic of the study was the parents’ fears and worries before and during clubfoot treatment. The Child Health Questionnaire and the American Academy of Orthopedic Surgeons Pediatric Outcomes Data Collection Instrument are two established measurement tools to describe the health status of children [20]. But as far as we know, there is no established questionnaire targeting the parents of children with pediatric orthopedic problems in the known literature. Therefore, we had to develop a questionnaire to determine parental distress.

The first section in our questionnaire related to the psychological and physical stress related to the child’s deformity. In the recent literature, it is accepted that parents of chronically impaired children, e.g., intellectual disability are limited in their psychological and physical health [21–23]. Therefore, we included the question of the parents’ current physical capacity and mental resilience in relation to the therapy of the child in our questionnaire.

Looking at the physical stress related to the child’s deformity, the treatment of clubfoot does not seem to disturb the daily life of the parents, unlike parents whose children have chronic diseases. From the authors’ point of view, this difference is due to the shorter duration of the exposure. In our study, most parents had only known about the deformity for a few days, so that no physical restrictions could develop yet.

In psychological stress our results indicate higher values, especially at the beginning of the treatment (*T*_0_). We observe a significant decrease in distress during treatment. In addition to the clinically visible decrease in clubfoot, the reasons for this can also be habituation effects to the weekly change of plaster and the deformity.

In another topic, we asked about the parents’ own interests (partnership, spare time, finance, professional life), because elevated marital conflicts and divorces in parents with chronically impaired children are reported in the literature [24]. In parents score, we also observed a slight distress of the parents, especially since the additional burden of the treatment is rather small in context with the changes in the daily life of the parents due to the care of the baby [25].

The child’s matters were divided in motion score and child score (general child concerns), in order to receive a more detailed result. In child score, we detected the highest score values without relevant decrease during therapy. We were able to detect high fears and worries when asking about teasing, school, and later careers. However, these fears seem essentially unfounded. Roye et al. found in their survey of 46 clubfoot patients that 81% were never teased because of their clubfoot and 19% sometimes [9]. From this data, it can be concluded that the risk of being teased for other reasons in adolescence (such as, e.g., weight-based teasing) is much higher [26].

Contemplating the motion score, we found also high values, declining during therapy. We expected this decrease as a result of the positive feedback for the parents regarding the clinically improving deformity during treatment. These prospects are supported by the good outcome of the Ponseti method, in many endpoints comparable to normal feet, as Church et al. showed in a follow-up examination of at least 5-year-old children [27]. In functional scores (e.g., PODCI (Pediatric Outcomes Data Collection Instrument)—transfer and basic mobility, pain, happiness) [28], no differences between clubfoot patients and the comparison group could be found.

The results of the survey in general showed small to medium concerns of the parents with a mean point score of maximum 3.11 (child score) and a minimum 1.44 (parents score). From the authors’ point of view, avoiding high point scores is primarily related to the informational discussion before the beginning of the study, in which the high success rate of conservative treatment according to Ponseti is explained in detail.

As a last important result, we found no correlation between the severity of clubfoot (Pirani score) and the own mental resilience. On the contrary, a study participant whose child had a rather low Pirani score with a point value of 3 showed a particularly high own mental resilience of 5. Looking at life-threatening diseases in children, such as cancer, there is a correlation between severity of the disease, number of complications, and parental distress (e.g., disease-related fear, anxiety, and depression) [29]. This effect could not be found in the lower stress situation of clubfoot treatment. This fact should be considered when dealing with affected parents in the daily routine and it should not be erroneously assumed that parents whose children have a discrete finding need less support.

### Study limitations

One limitation of our study is the lack of validation of the questionnaire. The validation must be done in further studies before the quality of the questionnaire can be finally reported. Furthermore, the questionnaire was originally created in German and translated for publication. Errors can also arise during the translation. Finally, the questionnaire is deliberately kept short for simple clinical use, which may have the consequence that less common parents’ concerns are occasionally omitted.

Nevertheless, the question of the present study on the worries and fears of parents during clubfoot treatment can be answered, from the authors’ point of view.

## Conclusion

Many practitioners in children’s orthopedic outpatient clinics deal with parents of children with clubfoot every day as part of the conservative treatment according to Ponseti. In addition to the correct practical implementation of the plaster casting, mental support of the parents is important from the authors’ point of view. In this study, the relevant parents’ worries and their course during treatment could be demonstrated. According to our results, particular emphasis should be placed on educating parents about the excellent long-term results in the function of the treated feet and the global scores (PODCI), especially as this topic shows the greatest fears and worries.

## Data Availability

The datasets used and/or analyzed during the current study are available from the corresponding author on reasonable request.
